# Downregulated circulating microRNAs after surgery: potential noninvasive biomarkers for diagnosis and prognosis of early breast cancer

**DOI:** 10.1038/s41420-018-0089-7

**Published:** 2018-08-06

**Authors:** Yaohui Wang, Wenjin Yin, Yanping Lin, Kai Yin, Liheng Zhou, Yueyao Du, Tingting Yan, Jinsong Lu

**Affiliations:** Department of Breast Surgery, Renji Hospital, School of Medicine, Shanghai Jiao Tong University, Shanghai, China

**Keywords:** Diagnostic markers, Breast cancer, Outcomes research, Prognostic markers, Diagnostic markers

## Abstract

Success in curing breast cancer largely depends on the stage at diagnosis. Circulating microRNAs are becoming a promising noninvasive biomarker. We postulate that a postoperative decline in circulating microRNAs might have diagnostic and prognostic value. Applying high-throughput microarrays, we screened the dysregulated microRNAs in paired serum samples before and after surgery. The relative concentrations of putative markers between the early breast cancer and cancer-free groups were evaluated in the training set and verified in the validation set. Sensitivity, specificity, and receiver operating characteristic (ROC) curves were used to assess diagnostic value. Survival analysis was performed using Kaplan–Meier estimates and a Cox proportional hazards model. Five microRNAs significantly reduced after surgery were selected for the training set. We found that miR-130b-5p, miR-151a-5p, miR-206, and miR-222-3p were significantly higher in the breast cancer group. Each of the four microRNAs had potential diagnostic value. The combined four microRNAs (training set: area under the curve (AUC) 0.8457; validation set: AUC 0.9309) had better diagnostic value than each single microRNA. MiR-222-3p was an independent prognostic factor for disease-free survival (HR = 13.19; 95% CI, 1.06–163.59; *P* = 0.045). Patients with no fewer than three highly expressed miRNAs had shorter DFS than patients with 0–2 highly expressed miRNAs (HR = 2.293; 95% CI, 1.128–0.662; *P* = 0.022). Our findings indicate that postoperatively downregulated circulating miR-130b-5p, miR-151a-5p, miR-206, and miR-222-3p may be potential biomarkers for breast cancer diagnosis and prognosis.

## Introduction

Breast cancer is the most commonly diagnosed cancer and the second leading cause of cancer death among females, accounting for 30% of all new cancer diagnoses and 21.2% of cancer deaths^1^. Fortunately, death rates have been declining in recent years, largely as a result of early diagnosis and improved treatment^2,3^. The 5-year survival rate of patients with early breast cancer is more than 90%, which is much higher than 20% for those with a primary diagnosis of metastatic breast cancer^4^. Therefore, early detection is an effective measure to improve the prognosis of breast cancer.

Mammography is a widely used tool for breast cancer screening. However, its application has become increasingly controversial due to its limitations of underdiagnosis of cancer patients with dense breasts and over-diagnosis of those who might not require surgery^5,6^. There is an urgent need for circulating biomarkers that might remedy mammography’s defects and pair with it in clinical practice. As we know, certain serum tumor markers associated with breast cancer, such as carbohydrate antigen 153 (CA153) and carcinoembryonic antigen (CEA), only apply to the monitoring of disease recurrence and not to the early diagnosis of breast cancer^7^. Therefore, the exploration of non-invasive biomarkers for early detection of breast cancer is a major challenge in the diagnosis and management of breast cancer.

MicroRNAs (miRNAs) are ∼22-nucleotide non-coding RNAs that play vital regulatory roles in triggering either translational repression or RNA degradation^8^. Aberrant expression of miRNAs is associated with many diseases, particularly most types of cancers^9^. MiRNAs are released into the blood and exist there stably, reproducibly, and consistently among individuals with the same disease condition^10^. Therefore, circulating miRNAs are becoming a promising, novel, non-invasive biomarker.

Until recently, it was widely recognized that circulating miRNAs come from the tumor itself^11,12^. Circulating nucleic acids are remarkably related to the growth, progression, metastasis, and histopathological characteristics of tumors^13^. Additionally, it was reported that the expression of specific circulating miRNAs is consistent with that of tumor tissues^14^, and the tumor might selectively secrete miRNAs into circulation^15^. Moreover, circulating miRNAs might be downregulated after certain effective treatments, such as chemotherapy^16^ or surgery^17^.

On these premises, we postulated that specific miRNAs secreted into circulation by the tumor would decrease after tumor resection, which might serve as a potential biomarker to track the tumor. Thus, our study was carried out by longitudinal comparison of miRNA profiles before and after surgery in individual patients. We aimed to investigate the diagnostic and prognostic value of the downregulated miRNAs after surgery in breast cancer.

## Results

### General characteristics

The baseline characteristics of the control group and breast cancer group in the training and validation set are listed in Table 1. The pathological characteristics of the breast cancer group were evenly balanced between the training and validation sets. There were no significant differences in the distributions of age or menstruation status between the control and breast cancer groups in either the training or validation databases (Supplementary Table S1).

**Table 1:** Baseline characteristics of study participants in the training and validation set

Variable	Training set		Validation set		*P*
	No.	%	No.	%	
Control group count	24		44		
*Age, years*					
≤50	13	54.17	25	56.82	0.833
>50	11	45.83	19	43.18	
*Menstruation*					
Pre-menopause	13	54.17	25	56.82	0.833
Post-menopause	11	45.83	19	43.18	
Breast cancer group count	24		58		
*Age, years*					
≤50	13	54.17	27	46.55	0.53
>50	11	45.83	31	53.45	
*Menstruation*					
Pre-menopause	13	54.17	29	50	0.731
Post-menopause	11	45.83	29	50	
*Histologic tumor size* (*cm*)					
≤2	9	37.5	23	39.66	0.711
2–5	12	50	31	53.45	
>5	3	12.5	4	6.89	
*Lymph nodes*					
Positive	16	66.67	30	51.72	0.215
Negative	8	33.33	28	48.28	
*Estrogen receptors*					
Positive	14	58.33	29	50	0.492
Negative	10	41.67	29	50	
*Progestogen receptors*					
Positive	14	58.33	29	50	0.492
Negative	10	41.67	29	50	
*HER2 status*					
Positive	12	50	24	41.38	0.474
Negative	12	50	34	58.62	
*Ki67 labeling index*					
≤14	1	4.17	6	10.34	0.362
>14	23	95.83	52	89.66	
*Histological grade*					
1	0	0	0	0	0.097
2	8	33.33	31	53.45	
3	16	66.67	27	46.55	

### Discovery phase

**Fig. 1:**
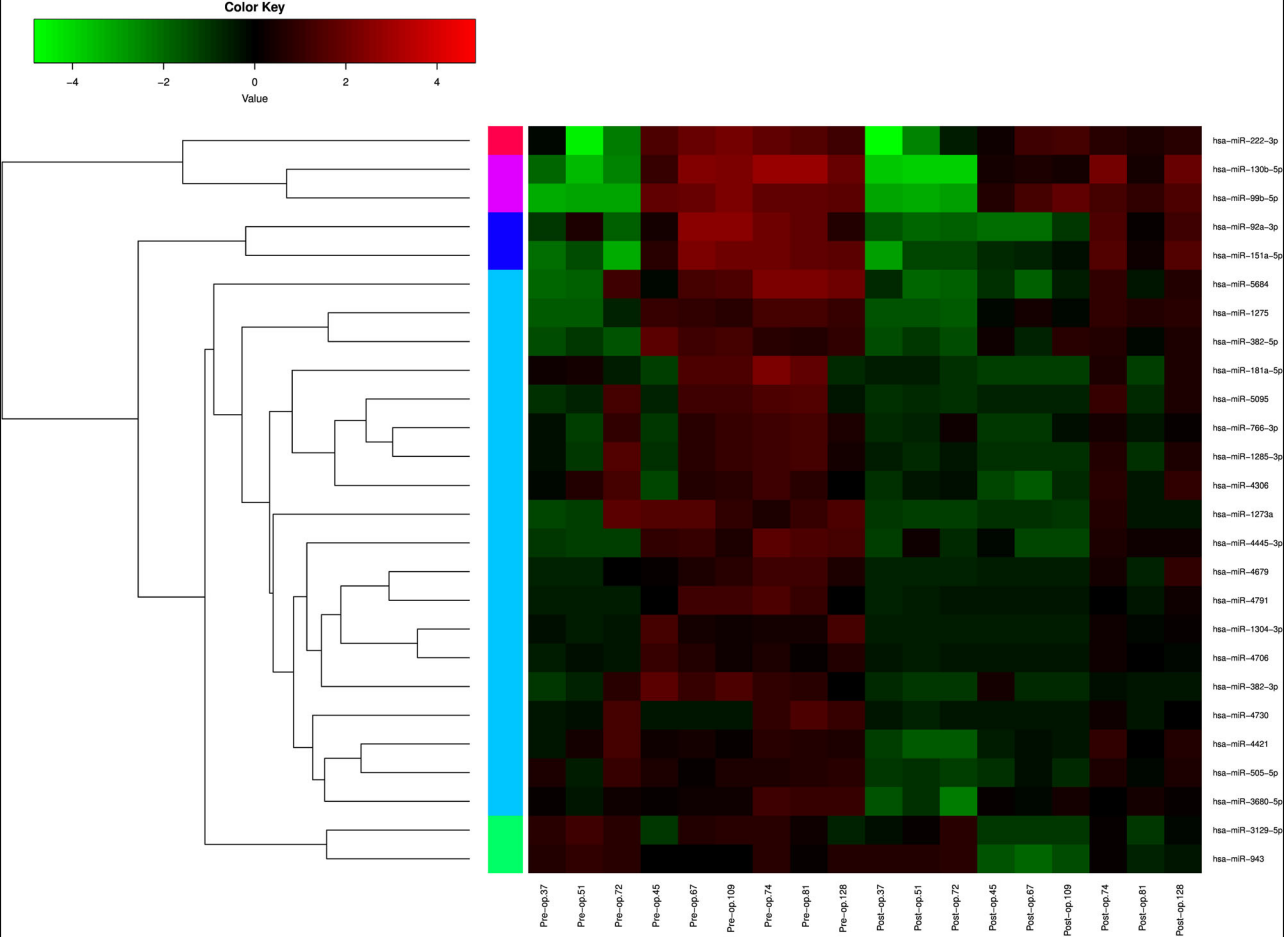
Heat map and hierarchical clustering of downregulated miRNA expression signatures compared postoperative with preoperative signatures in nine matched-pair breast cancer patients’ samples. Each row represents a miRNA, and each column represents a sample. The miRNA clustering tree is shown on the left. The color scale shown on the top illustrates the relative expression level of a miRNA in a certain slide: red color represents a high relative expression level and green color represents a low relative expression level. Those miRNAs whose fold change is more than 1.5 and *P* value less than 0.05 are listed here

Using high-throughput microarrays, 2037 miRNAs were identified. MiRNA expression was analyzed by comparing preoperative and postoperative samples in a total of nine paired samples. Furthermore, hierarchical clustering was performed to show distinguishable downregulated miRNA expression profiling after surgery (Fig. 1). After screening, we found that five circulating miRNAs (hsa-miR-130b-5p, hsa-miR-222-3p, hsa-miR-151a-5p, hsa-miR-943, hsa-miR-206) were selected for subsequent testing.

### Training phase

In the training set, we found that the expression levels of serum miR-130b-5p (*P* = 0.004), miR-151a-5p (*P* = 0.003), miR-206 (*P* = 0.0086), and miR-222-3p (*P* = 0.0146) were significantly higher in the breast cancer group than in the control group, while no difference was observed for miR-943 between the two groups (*P* = 0.3369; Supplementary Fig. S1).

**Fig. 2:**
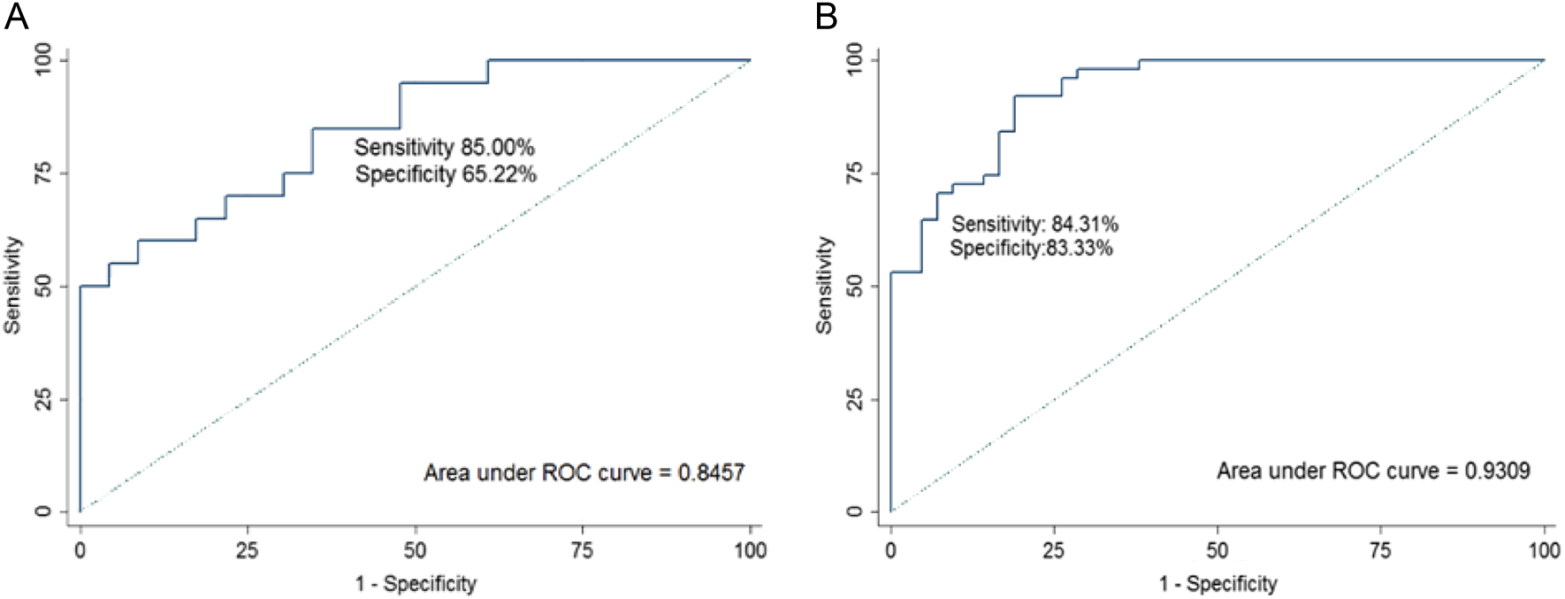
Receiver-operating characteristic (ROC) curve analysis of the four miRNAs for detecting breast cancer. (**A**) ROC curve analysis of combined miR-130b-5p,miR-151a-5p,miR-206 and miR-222-3p in the training set (AUC 0.8457,sensitivity 85.00%, specificity 65.22%). (**B**) ROC curve analysis of combined miR-130b-5p,miR-151a-5p,miR-206 and miR-222-3p in the validation set (AUC 0.9309,sensitivity 84.31%, specificity 83.33%).

MiR-130b-5p, miR-151a-5p, miR-206, and miR-222-3p were all worthy diagnostic markers (Supplementary Fig. S2). The AUC of miR-130b-5p was 0.7486 [95% confidence interval (CI): 0.6053–0.8918]. At the cut-off value of 2.48, the optimal sensitivity and specificity of miR-130b-5p were 60.87% and 86.96%, respectively. The AUC of miR-151a-5p was 0.7599 (95% CI: 0.6176–0.9023). At the cut-off value of 2.34, the optimal sensitivity and specificity of miR-151a-5p were 61.90% and 79.17%, respectively. The AUC of miR-206 was 0.7302 (95% CI: 0.5810–0.8793). At the cut-off value of 3.52, the optimal sensitivity and specificity of miR-206 were 52.38% and 87.50%, respectively. The AUC of miR-222-3p was 0.7066 (95% CI: 0.5540–0.8592). At the cut-off value of 1.58, the optimal sensitivity and specificity of miR-222-3p were 66.67% and 70.83%, respectively. The combined four miRNAs had better diagnostic value than each single miRNA (AUC 0.8457, sensitivity of 85.00%, specificity of 65.22%; Fig. 2A).

### Validation phase

The diagnostic values of these four highly expressed miRNAs in breast cancer (miR-130b-5p, miR-151a-5p, miR-206, miR-222-3p) were validated in the validation set (Supplementary Fig. S3). Their AUC values were 0.7276, 0.7959, 0.8605, and 0.8860, respectively (Supplementary Fig. S4). Each of the four miRNAs had potential value for diagnosis. The diagnostic accuracy of the combined four miRNAs was an AUC of 0.9309, a sensitivity of 84.31% and a specificity of 83.33% (Fig. 2B).

### Associations of the expression of the four miRNAs with the clinicopathological characteristics of the breast cancer patients

The expression levels of the circulating miRNAs in the breast cancer samples were compared with clinicopathologic variables (Supplementary Table S2). The expression level of serum miR-222-3p was significantly associated with tumor size (*P* = 0.0440).

### Prognostic value of the four miRNAs in breast cancer

**Fig. 3:**
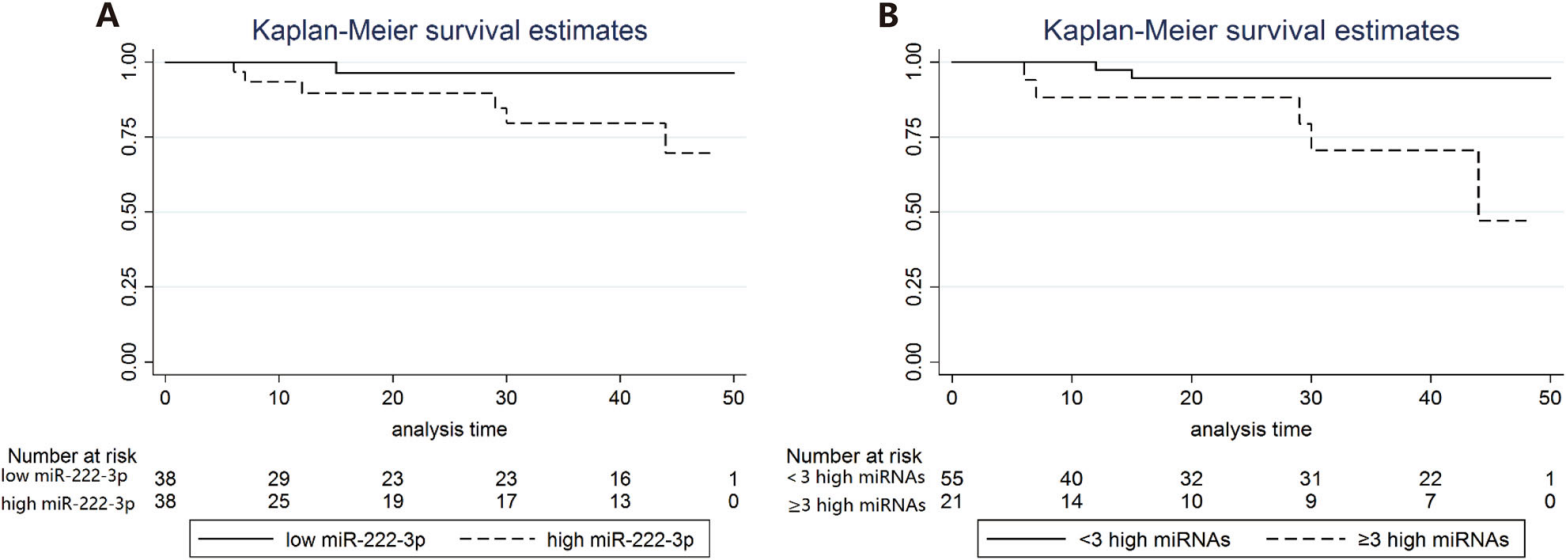
The Kaplan-Meier survival curve for disease-free survival in the breast cancer patients. (**A**) Analysis by the expression of miR-222-3p. (**B**) Analysis by the number of highly expressed miRNAs.

We prospectively analyzed the association of miRNA expression with disease-free survival (DFS) in breast cancer patients in both the training set and validation set. At the time of analysis, the median follow-up time was 31 months (2.58 years). MiR-222-3p was significantly associated with DFS. In the log-rank univariate survival analysis, DFS in the high expression group was significantly inferior to that in the low expression counterpart (log-rank *P* = 0.0386; Fig. 3A). In the multivariate survival analysis, miR-222-3p was an independent prognostic factor of DFS (HR = 13.19; 95% CI, 1.06–163.59; *P* = 0.045; Table 2). We did not find an independent prognostic value of miR-130b-5p, miR-151a-5p or miR-206 in either the univariate or multivariate survival analysis. On the other hand, the number of highly expressed miRNAs varied by patient. Patients with three or more highly expressed miRNAs had shorter DFS than patients with 0–2 highly expressed miRNAs (log-rank *P* = 0.0038; Fig. 3B; HR = 2.293; 95% CI, 1.128–0.662; *P* = 0.022; Table 3).

**Table 2:** Multivariate survival analysis of miR-222-3p expression and disease-free survival

Variable	HR	95% CI of HR	*P*
MiR-222-3p (low vs. high)	13.186	1.063–163.591	0.045
Age (≤50 vs. >50)	0.279	0.041–1.896	0.192
Histologic tumor size (≤2 cm vs. 2–5 cm vs. >5 cm)	0.228	0.050–1.030	0.055
No. of positive nodes (0 vs. 1–3 vs. ≥4)	1.402	0.399–4.931	0.598
Hormone receptor status (negative vs. positive)	0.547	0.071–4.227	0.564
HER2 status (negative vs. positive)	0.127	0.009–1.155	0.065
Grade (2 vs. 3)	5.052	0.691–36.946	0.111

*HR* hazard ratio, *CI* confidence interval, *HER2* human epidermal growth factor receptor 2

**Table 3:** Multivariate survival analysis of numbers of highly expressed miRNAs and disease-free survival

Variable	HR	95% CI of HR	P
No. of highly expressed miRNAs (<3 vs. ≥3)	2.293	1.128–4.662	0.022
Age (≤50 vs. >50)	0.397	0.069–2.301	0.303
Histologic tumor size (≤2 cm vs. 2–5 cm vs. >5 cm)	0.224	0.043–1.160	0.074
No. of positive nodes (0 vs. 1–3 vs. ≥4)	1.905	0.444–8.169	0.386
Hormone receptor status (negative vs. positive)	0.476	0.043–5.261	0.545
HER2 status (negative vs. positive)	0.353	0.025–5.011	0.442
Grade (2 vs. 3)	3.060	0.338–27.685	0.320

*HR* hazard ratio, *CI* confidence interval, *HER2* human epidermal growth factor receptor 2

### Functional analysis of miRNAs

In total, 1590 genes were predicted by at least two miRNA target databases, and 136 overlapped in three databases (Supplementary Fig. S5A). According to the GO analysis, target genes were divided into different kinds of biological processes, including positive regulation of cellular process and system development (Supplementary Fig. S5B). In the KEGG pathway analysis, it was predicted that four miRNAs might participate in many signal transduction pathways. The 10 most enriched signaling pathways were dorso-ventral axis formation, renal cell carcinoma, pathways in cancer, MAPK signaling pathway, estrogen signaling pathway, ras signaling pathway, erbB signaling pathway, rap1 signaling pathway, regulation of actin cytoskeleton, and proteoglycans in cancer (Supplementary Fig. S5C).

## Discussion

Circulating miRNAs are emerging as innovatively promising biomarkers for the detection of breast cancer at an early stage, prediction of prognosis, and monitoring of the effect of therapy^18^. This study focused on the potential diagnostic and prognostic role of the downregulated serum miRNAs of early breast cancer patients after tumor resection. In this study, we first identified that four serum miRNAs (miR-130b-5p, miR-151a-5p, miR-206, and miR-222-3p) decline after surgery, and this novel miRNA panel has promising diagnostic value as a set of non-invasive biomarkers for early breast cancer. To our knowledge, this is also the first time that the prognostic value of circulating miR-222-3p and the combined four miRNAs is highlighted for breast cancer.

The hypothesis that tumor-related biomarkers, such as antigens and miRNAs can be shed from tumor tissues or cells with ongoing disease and be present in blood is well accepted^19^. Chen et al. observed significant differences between miRNAs in the serum and blood cells of cancer patients but no differences between the serum and blood cells of healthy individuals^10^. The expression levels of some circulating miRNAs were downregulated when the tumor disappeared. Previously, miR-324-5p was detected in preoperative plasma and was downregulated or even undetectable 6 months after surgery^15^. Serum miR-155 was downregulated not only after surgery but also after chemotherapy^16^. We found four miRNAs (miR-130b-5p, miR-151a-5p, miR-206, and miR-222-3p) that were reduced after surgery in our study. To some extent, those miRNAs could prove the existence of a tumor.

If specific circulating miRNAs came from the solid tumor itself, the expression level in circulation might be partially consistent with the expression level in the tumor tissue. We found that serum miR-130b-5p was upregulated in all breast cancer patients. Consistent with our finding, miR-130b-5p was upregulated in triple-negative breast cancer tissue^20^, and miR-130b-3p was upregulated in ER-positive breast cancer tissue^21^ compared with adjacent normal tissue. Wu et al. found that miR-222 was upregulated in both serum and tissue in breast cancer patients^22^. This finding was in line with our results indicating that miR-222-3p was upregulated in the serum. Thus, this partially proved that circulating miR-130b-5p and miR-222-3p might come from the tumor and can be used as biomarkers for tracking the tumor.

Among the postoperatively downregulated miRNAs, several are known onco-miRNAs. MiR-130b promotes cell migration and invasion of the bladder cancer by regulating phosphatase and tensin homolog (PTEN)^23^ and accelerates the malignant progression of triple-negative breast cancer by mediating the repression of the cyclin G2 gene^20^. In ER-positive breast cancer, overexpression of miR-130b can induce multidrug resistance, which is tightly related to treatment failure and poor prognosis^21^. A tumorigenic role for miR-151a is suggested in non-small-cell lung cancer via its targeting of E-cadherin mRNA and induction of proliferation, migration, and partial epithelial-to-mesenchymal transition (EMT)^24^. Overexpressing miR-151a-5p can increase cell proliferation in the MCF-7 cell line (ER-positive breast cancer cell line) and the JIMT-1 and KPL-4 cell lines (ER-negative breast cancer cell lines)^25^. MiR-206 upregulation contributes to the abrogation of estrogenic responses in MCF-7 cells and a luminal to basal-like phenotypic switch^26^. MiR-221/222 was reported to play a critical role in the development and progression of breast cancer^27^, such as avoiding cell death from tumor suppressors^28^, monitoring angiogenesis^29^, and promoting the epithelial-to-mesenchymal transition^30^. Therefore, the crucial oncogenic roles can explain the poor prognostic value of the higher expression of the four miRNAs in our study.

Several studies have shown that dysregulated expression of miRNAs might be associated with clinicopathological features, such as lymph node metastasis, tumor stage, and clinical outcomes^31,32^. At the tissue level, the high expression of miR-222 was related to high T stage, a high histological grade, a high Ki-67 proliferation index and endocrine therapy resistance^33^. Correspondingly, at the circulation level, we also found that miR-222-3p was significantly associated with tumor size. Consequently, the relationships between miR-222 and aggressive clinicopathological features might account for the prognostic role of miR-222. High tissue miR-222 was associated with poor DFS in hormone receptor-positive breast cancer in a previous study^33^. Our result first confirmed that serum-miR-222-3p was an independent prognostic factor for DFS in all breast cancer patients. In our analysis, circulating miR-222-3p was proved to be not only a potential diagnostic biomarker but also a promising prognostic biomarker.

The potential limitations of this study were as follows. First, the sample size was relatively small. Large-scale validation analyses are needed to confirm the diagnostic value of these four circulating miRNAs for breast cancer. Second, we hypothesized that the downregulated miRNAs after surgery were released by the tumor and might indicate the existence of a tumor. However, our team did not test the expression of these miRNAs in the tumor or its adjacent tissues. Third, the follow-up periods tended to be relatively short.

In conclusion, the current study demonstrated the potential roles of postoperatively downregulated serum miR-130b-5p, miR-151a-5p, miR-206, and miR-222-3p as non-invasive biomarkers for early breast cancer. The combination of four miRNAs might have better accuracy, sensitivity, and specificity. MiR-222-3p may be a promising biomarker for the early detection and prognosis prediction of breast cancer.

## Materials and methods

### Patients

Patients were prospectively selected from those diagnosed as breast cancer and undergoing surgery between July 2012 and December 2013. Main eligibility criteria included female sex, age ranging from 18 to 75 years old, and an initial diagnosis of primary breast cancer without distant metastases. Exclusion criteria included neoadjuvant treatment before surgery, pregnancy or lactation, and previous history of any malignancy. Ethical approval was granted by the Ethics Committee of Fudan University Shanghai Cancer Center (FUSCC). Written informed consent was obtained from each participant in this study.

### Study design

The study was designed into four stages.

The first stage was the discovery phase, which identified the differential expression of circulating miRNAs between preoperative and postoperative serum in early-stage breast cancer. Applying high-throughput microarrays, we screened the dysregulated circulating miRNAs in paired serum samples of nine patients (T_1-3_N_0-3_M0) before and after surgery. In these patients, three subtypes [estrogen receptor (ER) and/or progesterone receptor (PR) positive and human epidermal growth factor receptor-2 (HER2) negative, ER and PR negative and HER2 positive, triple-negative) were evenly distributed at a 1:1:1 ratio (three patients for each subtype). Compared to the preoperative level, postoperative circulating miRNAs that declined obviously with *P* value < 0.05 and the fold change ≥1.5 were selected. Furthermore, selected miRNAs were screened through literature review and our prestudy results. Then, six circulating miRNAs discovered via microarrays were candidate targets for further evaluation.

The second stage was the training phase, which tested the differential preoperative expression of candidate circulating miRNAs between breast cancers and non-malignant controls (patients with benign breast diseases and healthy women) by quantitative real-time polymerase chain reaction (qRT-PCR). Initially, six miRNAs screened via the first phase were tested in 24 breast cancers and 24 controls by qRT-PCR to explore the diagnostic value of significantly variant miRNAs. Consequently, four miRNAs were found to be differentially expressed between breast cancers and controls and entered the next stage.

The third stage was the validation phase, which confirmed the diagnostic value of the four miRNAs in another database with more independent serum samples. The four miRNAs obtained via the second phase were tested in 58 breast cancers and 44 controls. The diagnostic performance of the selected miRNA panel was finally validated.

The fourth stage explored the prognostic value of these miRNAs through prospective follow-up of all breast cancer patients.

### RNA extraction

RNA was isolated from 0.5 mL of serum by using the mirVana PARIS Kit (Ambion, TX, United States). The whole procedure was performed following the instructions of the kit. The concentration and integrity of RNA were quantified with a NanoDrop ND-1000 (Thermo Scientific, Wilmington, United States).

### Microarray

We used miRCURY^TM^ LNA expression arrays (v.18.0) from the Exiqon company in the high-throughput screen stage. Total RNA for the microarrays was harvested using TRIzol (Invitrogen, Carlsbad, United States) and the miRNeasy mini kit (Qiagen, Germany) according to the manufacturer’s instructions. After having passed RNA quantity measurement using the NanoDrop 1000, the samples were labeled using the miRCURY^TM^ Hy3^TM^/Hy5^TM^ Power labeling kit and hybridized on the miRCURY^TM^ LNA Array (v.18.0). The slides were scanned using the Axon GenePix 4000B microarray scanner. Scanned images were then imported into GenePix Pro 6.0 software (Axon) for grid alignment and data extraction. Replicated miRNAs were averaged, and miRNAs with intensities ≥30 in all samples were chosen for calculating the normalization factor. Expressed data were normalized using the median normalization. After normalization, significantly differentially expressed miRNAs were identified through Volcano Plot filtering.

### qRT-PCR

Quantification of candidate miRNAs in both the training phase and validation phase was determined by qRT-PCR using TaqMan MiRNA Reverse Transcription Kit (Ambion, TX, United States), TaqMan miRNA assays (Ambion, TX, United States), and TaqMan Universal PCR Master Mix (Ambion, TX, United States).

### Statistical analysis

For microarray analysis and qRT-PCR data analysis, the Mann–Whitney unpaired test was used for the comparison between breast cancers and controls. Sensitivity, specificity, and receiver operating characteristics (ROC) curves were used to assess the diagnostic value of circulating miRNAs. Area under the ROC curve (AUC) was used as an accuracy index for evaluating the diagnostic performance of the selected miRNA panel and each miRNA. We assessed the correlation between clinical characteristics and the preoperative expression of miRNAs at the diagnosis of breast cancer by using Wilcoxon rank sum test for two categorical independent variables and Kruskal–Wallis rank test for multiple categorical independent variables. DFS was defined as the time from surgery until first occurrence of locoregional relapse, contralateral breast cancer, distant metastasis, second primaries, and death from any cause. Patients who remained alive and disease-free at their date of last follow-up were censored. To calculate the relationship between DFS and miRNA expression, we dichotomized each miRNA expression value according to its median and classified the participants into the high-specific and low-specific miRNA expression group, respectively. The Kaplan–Meier method and the log-rank test were used to estimate DFS. Multivariate Cox proportional hazards models were adjusted for histologic tumor size (≤2; 2–5; >5 cm), number of positive lymph nodes (negative; 1–3 positive; ≥4 positive), age (≤50 years; >50 years), hormone receptor status (positive; negative), HER2 status (positive; negative), and grade of tumor (2; 3). Hazard ratios (HRs) with 95% CIs were obtained from Cox proportional hazards regression models, with HR <1 favoring the low expression of miRNAs. All *P* values were two sided. We used statistical software STATA 14.0 (Stata Corporation, College Station, TX, USA) for all statistical analyses and curves.

### Functional analysis of miRNAs

To explore the putative target genes of significantly differently expressed miRNAs, three miRNA target prediction databases were used: Targetscan (https://www.targetscan.org/vert_60/), Microcosm (http://www.ebi.ac.uk/enright-srv/microcosm/htdocs/targets/v5/), and Miranda (http://www.microrna.org/microrna/home.do). Final results were derived from the overlapping part of at least two databases. The gene ontology (GO; http://www.geneontology.org) and the Kyoto Encyclopedia of Genes and Genomes (KEGG) pathway analyses were applied for functional annotating and pathway enrichment analysis of the target genes.

## Electronic supplementary material

Supplementary Table S1

Supplementary Table S2

Supplementary Fig.S1

Supplementary Fig.S2

Supplementary Fig.S3

Supplementary Fig.S4

Supplementary Fig.S5

Supplementary figure legends

## Notes

**Supplementary Information** The online version of this article (10.1038/s41420-018-0089-7) contains supplementary material, which is available to authorized users.
