# Supplementary material for: Downregulated circulating microRNAs after surgery: potential noninvasive biomarkers for diagnosis and prognosis of early breast cancer
Source: Cell Death Discov. 2018 Aug 6;4:87. doi: 10.1038/s41420-018-0089-7 (PMC6078958; doi:10.1038/s41420-018-0089-7)
Supplement: Supplementary file 1 — Supplementary Table S1 [file 41420_2018_89_MOESM1_ESM.docx]

**Table S1. Baseline characteristics of study participants in the training and validation set between the breast cancer group and control group.**

|  | | **Training set** | | | | | | **Validation set** | | | |  |
| --- | --- | --- | --- | --- | --- | --- | --- | --- | --- | --- | --- | --- |
| **Characteristic** | **Control**  **Group** | | | | **Breast cancer group** | |  | **Control**  **group** | | **Breast cancer group** | |  |
|  | **No.** | | | **%** | **No.** | **%** | ***P*** | **No.** | **%** | **No.** | **%** | ***P*** |
| **Age, years** | | |  |  |  |  |  |  |  |  |  |  |
| ≥50 | | | 11 | 45.83 | 11 | 45.83 | 1 | 19 | 43.18 | 31 | 53.45 | 0.304 |
| < 50 | | | 13 | 54.17 | 13 | 54.17 |  | 25 | 56.82 | 27 | 46.55 |  |
| **Menstruation** | | |  |  |  |  |  |  |  |  |  |  |
| Post-menopause | | | 11 | 45.83 | 11 | 45.83 | 1 | 19 | 43.18 | 29 | 50 | 0.494 |
| Pre-menopause | | | 13 | 54.17 | 13 | 54.17 |  | 25 | 56.82 | 29 | 50 |  |
