# Supplementary material for: Downregulated circulating microRNAs after surgery: potential noninvasive biomarkers for diagnosis and prognosis of early breast cancer
Source: Cell Death Discov. 2018 Aug 6;4:87. doi: 10.1038/s41420-018-0089-7 (PMC6078958; doi:10.1038/s41420-018-0089-7)
Supplement: Supplementary file 2 — Supplementary Table S2 [file 41420_2018_89_MOESM2_ESM.docx]

**Table S2. Associations between circulating miRNA expression and clinicopathologic variables in breast cancer.**

| **Variable** | **Test** | **MiR-130b-5p**  ***P*-value** | **MiR-151a-5p**  ***P*-value** | **MiR-206**  ***P*-value** | **MiR-222-3p**  ***P*-value** |
| --- | --- | --- | --- | --- | --- |
| **Age**  **(≤50 vs >50)** | Wilcoxon rank sum test | *P*=0.3032 | *P*=0.6391 | *P*=0.6428 | *P*=0.8285 |
| **Menstruation**  **(Post-menopause**  **vs Pre-menopause)** | Wilcoxon rank sum test | *P*=0.4757 | *P*=0.3486 | *P*=0.5009 | *P*=0.9846 |
| **Histologic tumor size (≤2cm vs 2-5cm vs ＞5cm)** | Kruskal-Wallis rank test | *P*=0.9625 | *P*=0.8509 | *P*=0.9806 | ***P*=0.0440^*^** |
| **No. of positive nodes (Negative vs Positive)** | Wilcoxon rank sum test | *P*=0.4517 | *P*=0.3705 | *P*=0.7378 | *P*=0.8381 |
| **Estrogen receptors (Negative vs Positive)** | Wilcoxon rank sum test | *P*=0.4757 | *P*=0.8210 | *P*=0.3488 | *P*=0.9392 |
| **Progesterone receptors (Negative vs Positive)** | Wilcoxon rank sum test | *P*=0.5300 | *P*=0.9269 | *P*=0.2796 | *P*=0.5697 |
| **HER2 status**  **(Negative vs Positive)** | Wilcoxon rank sum test | *P*=0.9451 | *P*=0.3311 | *P*=0.3830 | *P*=0.1390 |
| **Grade**  **(II vs III)** | Wilcoxon rank sum test | *P*= 0.3741 | *P*=0.7408 | *P*=0.5132 | *P*=0.3764 |
| **Subtype**  **(Luminal like**  **vs HER2-positive**  **vs Triple-negative)** | Kruskal-Wallis rank test | *P*=0.6612 | *P*=0.5457 | *P*=0.5942 | *P*=0.8809 |
