# Supplementary figures and images for: Downregulated circulating microRNAs after surgery: potential noninvasive biomarkers for diagnosis and prognosis of early breast cancer
Source: Cell Death Discov. 2018 Aug 6;4:87. doi: 10.1038/s41420-018-0089-7 (PMC6078958; doi:10.1038/s41420-018-0089-7)

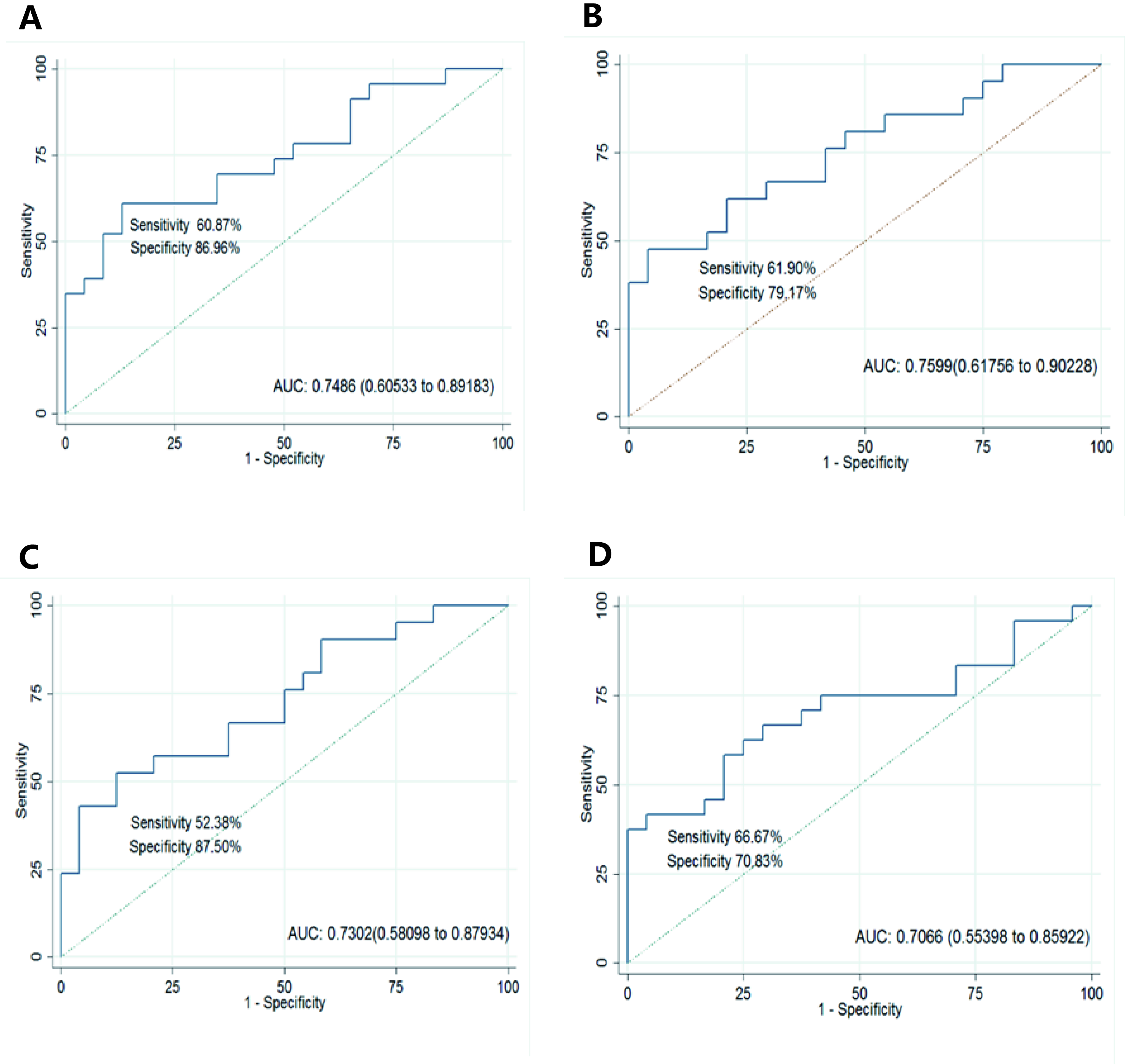

Supplement: Supplementary file 4 — Supplementary Fig.S2 [file 41420_2018_89_MOESM4_ESM.tif]

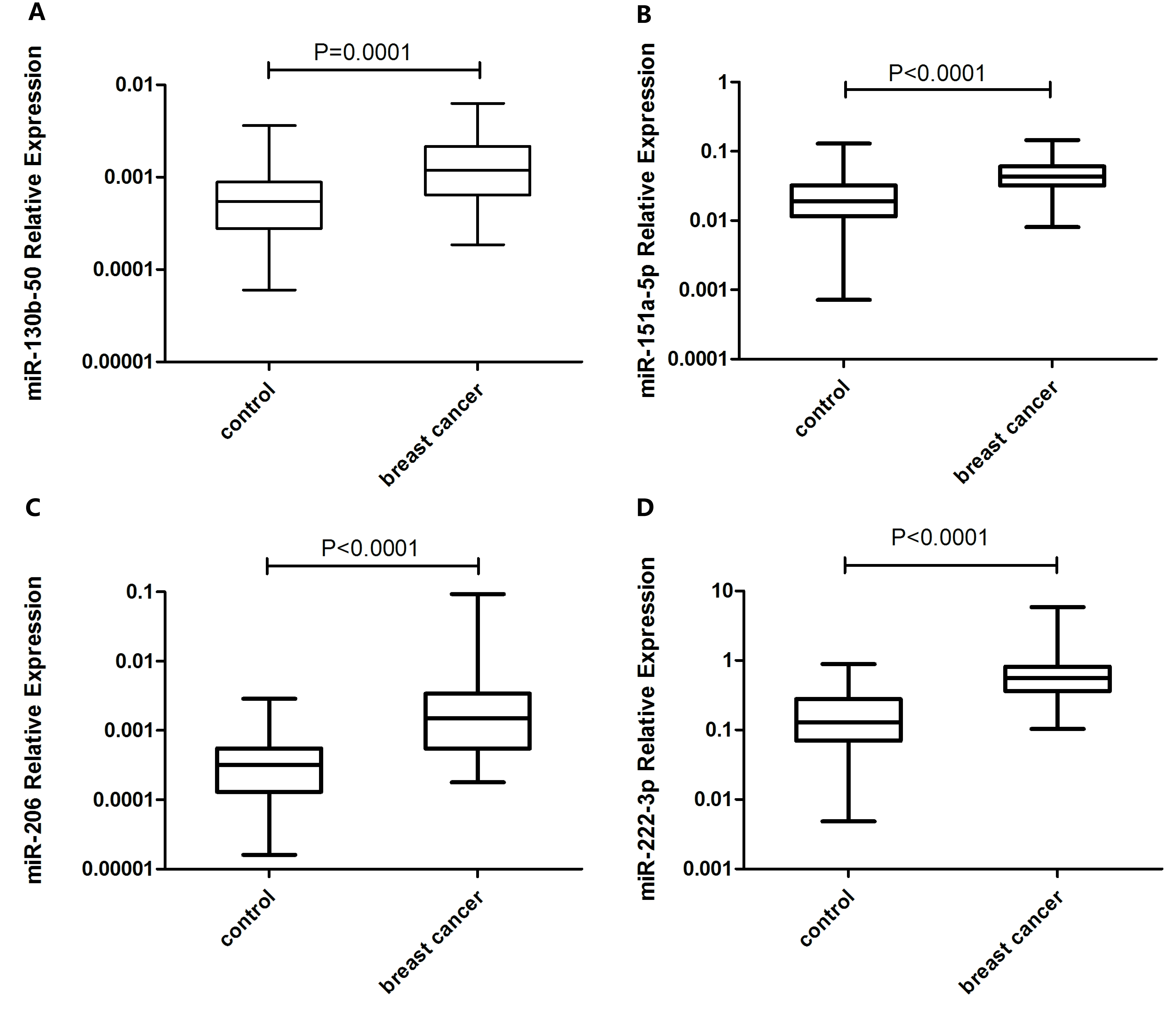

Supplement: Supplementary file 5 — Supplementary Fig.S3 [file 41420_2018_89_MOESM5_ESM.tif]

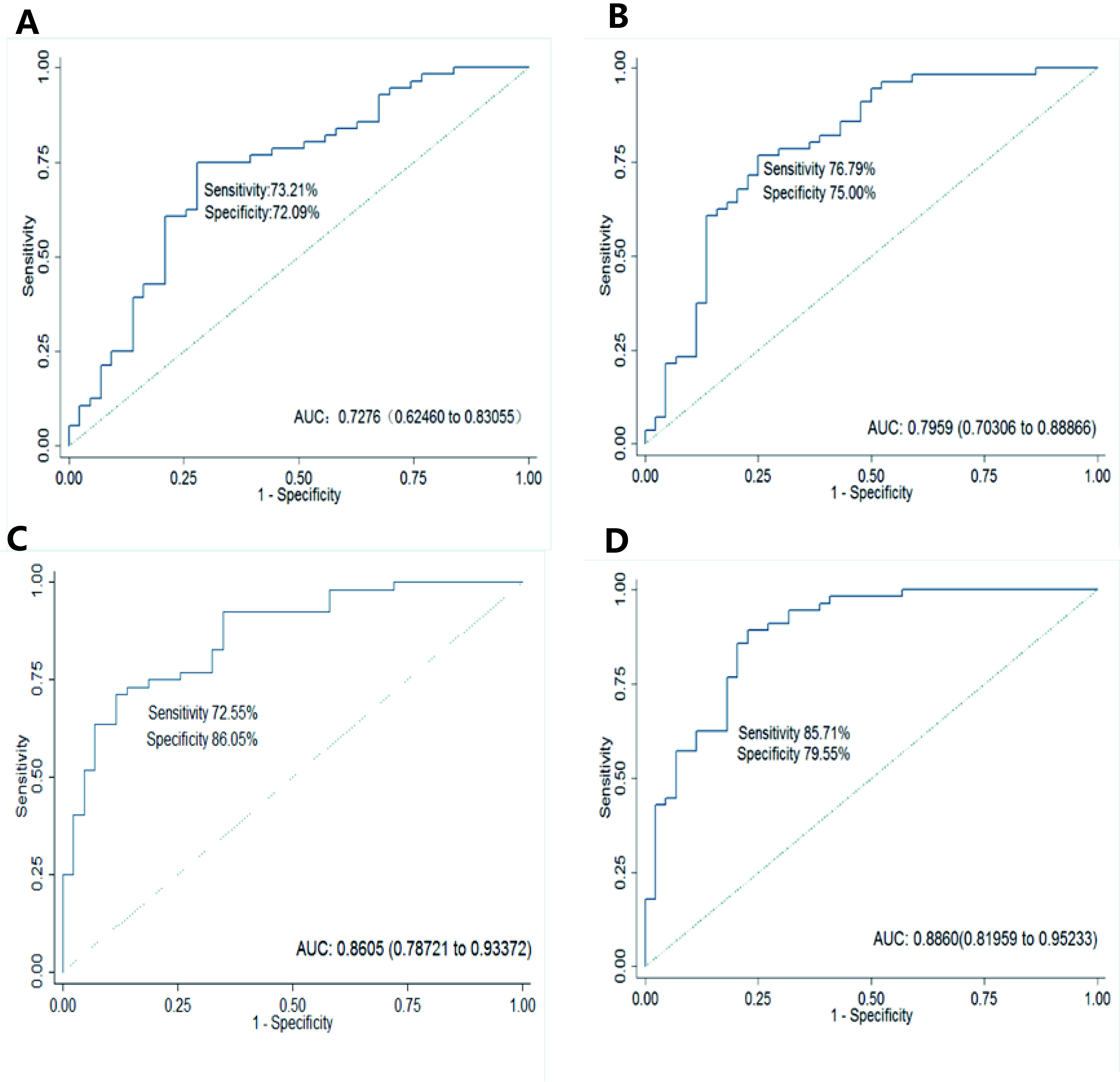

Supplement: Supplementary file 6 — Supplementary Fig.S4 [file 41420_2018_89_MOESM6_ESM.tif]

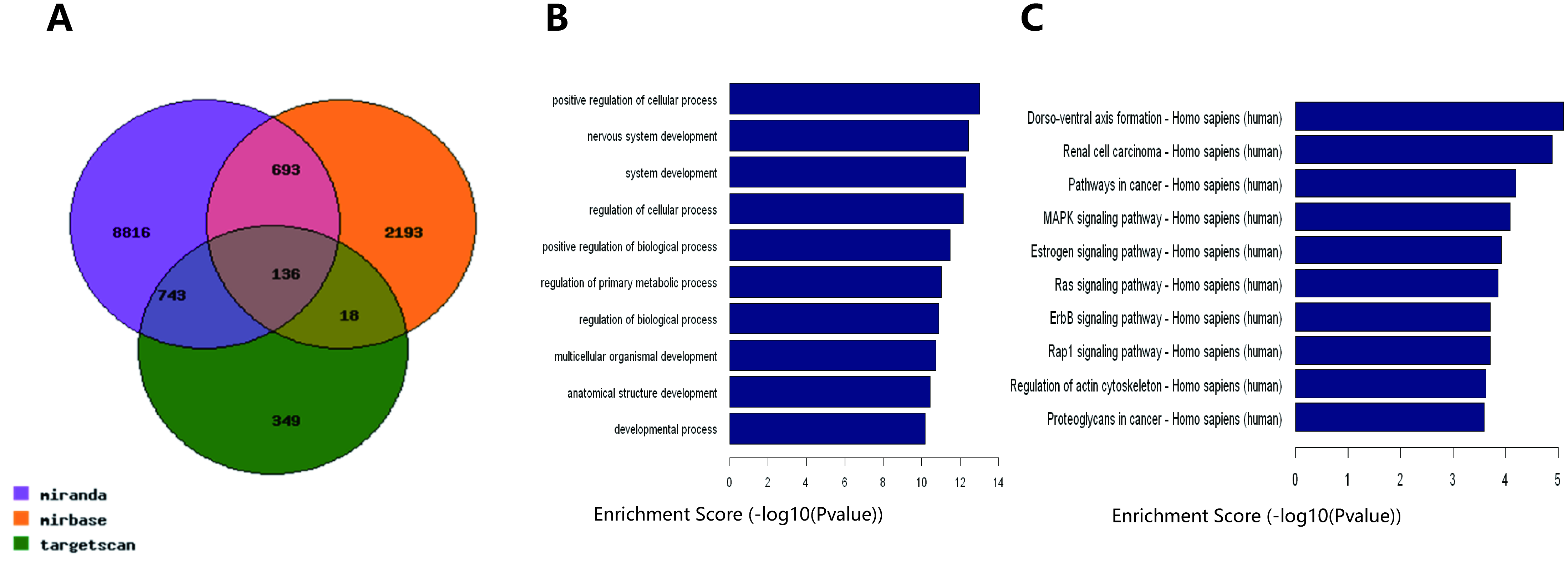

Supplement: Supplementary file 7 — Supplementary Fig.S5 [file 41420_2018_89_MOESM7_ESM.tif]
