# Supplementary material for: Downregulated circulating microRNAs after surgery: potential noninvasive biomarkers for diagnosis and prognosis of early breast cancer
Source: Cell Death Discov. 2018 Aug 6;4:87. doi: 10.1038/s41420-018-0089-7 (PMC6078958; doi:10.1038/s41420-018-0089-7)
Supplement: Supplementary file 8 — Supplementary figure legends [file 41420_2018_89_MOESM8_ESM.docx]

**Supplementary Figure Legends**

**Fig. S1 The relative expression of miR-130b-5p (A), miR-151a-5p (B), miR-206 (C), miR-222-3p (D) and miR-943 (E) in the breast cancer group and control group in the training set.**

**Fig. S2 Receiver-operating characteristic (ROC) curve analysis of miR-130b-5p (A), miR-151a-5p (B), miR-206 (C), and miR-222-3p (D) in the training set for detecting breast cancer.**

**Fig. S3 The relative expression of miR-130b-5p (A), miR-151a-5p (B), miR-206 (C) and miR-222-3p (D) in the breast cancer group and control group in the validation set.**

**Fig. S4 Receiver-operating characteristic (ROC) curve analysis of miR-130b-5p (A), miR-151a-5p (B), miR-206 (C), miR-222-3p (D) in the validation set for detecting breast cancer.**

**Fig. S5 Functional analysis of miRNAs. The putative target genes of significantly differently expressed miRNAs through three miRNA target prediction databases, including Targetscan (https://www.targetscan.org/vert_60/), Microcosm (http://www.ebi.ac.uk/enright-srv/microcosm/htdocs/targets/v5/) and Miranda (http://www.microrna.org/microrna/home.do) (A). Biological process analysis screened by GO analysis for four significant differentially expressed miRNAs (miR-130b-5p, miR-151a-5p, miR-206, miR-222-3p).** This showed the top ten Enrichment Score [-log10 (P value)]) values of the significant enrichment terms **(B)**. **KEGG signaling pathway analysis of** **four significantly differentially expressed miRNAs (miR-130b-5p, miR-151a-5p, miR-206, miR-222-3p).** This showed the top ten enrichment score [(-log10 (P value)) values of the significant enrichment pathway **(C).**

**Supplementary Table Legends**

**Table S1. Baseline characteristics of study participants in the training and validation set between the breast cancer group and control group.**

**Table S2. Associations between circulating miRNA expression and clinicopathologic variables in breast cancer.**
